# Special Issue “Mesenchymal Stromal Cells’ Involvement in Human Diseases and Their Treatment”

**DOI:** 10.3390/ijms25021269

**Published:** 2024-01-20

**Authors:** Mirjana Jerkic, Razieh Rabani

**Affiliations:** 1 The Keenan Research Centre for Biomedical Science of St. Michael’s Hospital, Unity Health Toronto, University of Toronto, Toronto, ON M5B 1T8, Canada; 2 CReATe Fertilty Center, Toronto, ON M5G 1N8, Canada; razieh.rabani@utoronto.ca

Mesenchymal stromal cells (MSCs) are multipotent, non-hematopoietic cells that have the ability to differentiate into several mature cell types, including adipocytes, chondrocytes, osteoblasts, and myoblasts [1]. However, in the literature, the terms mesenchymal stem and mesenchymal stromal cells are used, sometimes interchangeably, prompting the International Society for Cell & Gene Therapy to issue clarification of these terms (the ISCTs MSC committee criteria) [2].

MSCs and/or MSC-derived extracellular vesicles (EVs) hold significant potential as a therapeutic tool, by exercising both protective and regenerative effects on damaged tissue directly and indirectly, through immunomodulation, paracrine effects, and microvesicular and mitochondrial transfer [3,4,5]. The importance of mitochondrial transfer for MSC efficacy has been recently recognized. In this issue, Mukkala et al. [5] reviewed the literature on this topic and pointed out that MSCs enhance mitochondrial functions in the injured tissues by regulating mitochondrial quality control (MQC), including mitochondrial biogenesis, mitophagy, fission, and fusion. Also, both MSCs and injured cells have important roles in mitochondrial function improvements. Damaged somatic cells release mitochondria (Mt) that are recognized by MSCs as danger-signaling organelles. Engulfment and degradation of these Mt by MSCs enhance both expression of reparatory factors and mitochondrial biogenesis in the MSCs. Therefore, the capacity of MSCs to donate their own Mt to injured cells increases, boosting the Mt function and energy production of the recipient cells. All these events eventually lead to the improvement and regeneration of damaged tissues.

In order to enhance MSC therapeutic efficacy in injured tissues, efforts should be directed to MSC priming (i.e., activation or licensing) prior to exposure to a harmful microenvironment. Additionally, determining the appropriate dosage, timing, and administration route is crucial to ensure optimal outcomes in specific disorders.

Several abovementioned key questions regarding the MSC efficacy were tackled in this issue. Lee and Kwok [6] shed a light on genetic engineering techniques, priming 3D spheroid cell culture approaches that have the potential to significantly enhance the therapeutic potential of MSCs and MSC-EVs, especially in a context of systemic rheumatic diseases and their refractory forms. De Oliveira et al. [7] tested if the anti-inflammatory properties of MSCs could alleviate osteoarthritis (OA) in a pre-clinical rat model of OA. They found that a single injection of MSCs, regardless of administration route, did not effectively inhibit articular cartilage degeneration. The study reiterates the unresolved need to investigate the appropriate timing, dosage, and frequency of MSC therapy within specific conditions. Specifically, in the context of OA treatment, it may be essential to consider long-term protocols and periodic injections of MSCs in order to improve the therapeutic outcome [8,9].

Byrnes et al. [10] explored the MSC dosage regiment in pneumonia caused by antimicrobial-resistant (AMR) bacteria, such as Klebsiella (K). Treatment with a single dose of naïve MSCs appeared to be largely ineffective in rat pneumosepsis, whereas two doses of MSCs effectively improved lung function and reduced both bacterial load and lung tissue injury. Additionally, cytomix-licensed, i.e., activated MSCs exhibited superior performance when compared to naïve MSCs. Therefore, this clinically relevant model of pneumosepsis provided evidence that repeated doses of MSCs, particularly pre-activated MSCs, enhance their potential and treatment benefits.

Blanco et al. [4] compared the effect of MSC-EVs derived from various sources on polymicrobial sepsis in mice. Their findings revealed that treatment with EVs derived from bone marrow (BM) MSCs was more effective compared to MSC-EVs from other sources (adipose tissue or lungs), potentially related to observed differences detected in their proteome.

The interplay between MSCs and monocytes/macrophages represents one of the key mechanisms by which MSCs exert their therapeutic benefits [11,12,13]. Cortés-Morales et al. [14] investigated this interaction in a context of myocardial infarction (MI). They reported that co-culturing of macrophages (Mϕs), isolated from patients suffered from MI, with BM-MSCs led to the attenuation of the inflammatory activity of M1 Mϕs and enhancement of the activity of M2 Mϕs. Therefore, the application of MSC therapy in MI patients could improve cardiac tissue repair, as the regeneration and healing process is mainly driven by alternatively activated (M2-like) Mϕs [15].

MSC dysfunction could also be involved in disease pathobiology [16]. A great example of such a disease is acquired aplastic anemia (AA) [17]. MSCs provide a supportive environment for hematopoietic stem cells (HSCs) in the BM and coordinate their trafficking [18]. Therefore, MSC dysfunction could be involved in BM microenvironment impairment, leading to acquired AA. According to small number of clinical studies conducted so far, the infusion of MSCs, whether administered as a single dose or multiple times, could not effectively reconstitute the hematopoiesis in patients with AA. However, the combined treatment of MSCs along with HSC transplantation may prove to be more effective in AA than either treatment alone. As summarized in the review paper by Wang et al. [19], several studies have shown advantage of co-transplantation of MSC and HSC in AA, in both pediatric [20] and adult patients [21] with severe AA. Benefits of this co-transplantation, over HSC transplantation alone, include better hematopoietic reconstitution, reduced incidence of graft rejection, and an overall increase in patient survival rate. 

MSCs interaction with the microenvironment is also crucial for their therapeutic effects. However, in some circumstances, MSCs could even accelerate disease progression. This is notably observed in the utilization of MSCs for cancer treatment. Although immunomodulatory action of MSCs plays a major role in cancer cell therapy, the interactions between MSCs and the tumor microenvironment (TME) could be ambiguous, making the outcomes of MSC use for cancer treatment double-edged [22]. This controversial action of MSCs in TME is clearly reflected in the paper by Waltera et al., published in this issue [23]. The interactions of BM-MSCs with the tumor cells from the head and neck region resulted in elevated MSC secretion of MMP-9 and increased tumor invasiveness. At the same time, tumor cells reduced their MMP-9 promoter activity. Therefore, the interaction between MSCs and cancer cells is complex. More mechanistic insights are needed to fully understand this interplay, which will enable the development of novel, safe, and efficient treatment options and therapeutic strategies.

In summary, this issue represents a step forward in our knowledge about the effects of MSCs in various diseases and addresses certain gaps in understanding the mechanisms of MSC action in injured tissue. Beneficial actions of MSCs in different disorders are underlined, but it is also noted that, in some circumstances, the impact of MSCs could be detrimental. The main obstacles for regular clinical use of MSCs have to be solved, encompassing challenges such as cell heterogeneity, immunogenicity, culture conditions, dosage, treatment regimen, and more comprehensive understanding of MSC functions and their interactions with the specific microenvironment. Clinical trials conducted so far are promising. However, large-scale randomized trials with mechanistic studies conducted on patient samples are required for better understanding of MSC/MSC-EV interactions with injured cells. The results from these studies will offer the opportunity and knowledge for the improvement of cell therapy and for the development of novel treatment strategies, personalized approaches, and adequate MSC uses in a variety of disorders.
